# Refractive error and vision related quality of life

**DOI:** 10.1186/s12886-024-03350-8

**Published:** 2024-02-22

**Authors:** Mojtaba Rajabpour, Haleh Kangari, Konrad Pesudovs, Masoud Khorrami-nejad, Saeed Rahmani, Sahar Mohaghegh, Shima Moradnejad

**Affiliations:** 1 Department of Optometry, Faculty of Rehabilitation, Shahid Beheshti University of Medical Sciences, Imam Hossein Square, Damavand Avenue, Opposite to Bouali Hospital, Tehran, 1616913111 Iran; 2 School of Optometry and Vision Science, Medicine & Health, University of New South Wales, Kensington, NSW Australia; 3 Translational Ophthalmology Research Center, Farabi Eye Hospital, Tehran University of Medical Sciences, Tehran, Iran; 4 Optometry Department, School of Rehabilitation, Tehran University of Medical Sciences, Tehran, Iran; 5 Department of Health Promotion and Education, School of Public Health, Tehran University of Medical Sciences, Tehran, Iran

**Keywords:** Myopia, Astigmatism, Hyperopia, Refractive errors, Quality of life

## Abstract

**Background:**

To investigate and compare the vision-related quality of life (QOL) in different types of refractive error (RE).

**Methods:**

This cross-sectional study was performed on 200 subjects, categorized into four groups of 50 each, consisting of subjects with myopia, hyperopia, astigmatism, and emmetropia, the latter being the control group. The mean age of the participants was 23.88 ± 5.87 (range, 15 to 38: 110 females and 90 males). RE was defined as myopia, spherical equivalent (SE) < -0.25 diopters (D), hyperopia, SE > + 0.25 D, astigmatism, cylinder < -0.25 D, and emmetropia (-0.25 ≤ SE(D) ≤ + 0.25, cylinder ≥ -0.25). Groups are subdivided into very low magnitudes of RE (0.50 and 0.75) and significant RE (1.00 ≤). Vision-related QOL was assessed using the Persian version of the 25-item National Eye Institute Visual Functioning Questionnaire (NEI-VFQ-25). The NEI-VFQ was scored as visual function and socioemotional scales using Rasch analysis.

**Results:**

Corrected myopia, astigmatism, uncorrected myopia, and hyperopia had a lower vision-related QOL than emmetropes. (*P* < 0.001). Vision-related QOL in myopic subjects was lower than that in astigmatic participants. Very low myopes, who often do not use correction, had a significantly lower QOL than other groups.

**Conclusion:**

Individuals with refractive errors experience a lower QOL score than those without. Notably, the adverse impact on QOL score is significantly greater in myopic cases, particularly very low myopia, compared to other refractive errors. Therefore, it is strongly recommended not to neglect managing very low myopia since it may improve participants’ QOL.

## Background

Refractive error (RE), based on the International Classification of Diseases, is defined as a defect in focusing light on the retina that causes blurring of vision [1]. RE can be divided into three categories based on the location of the light focus: 1- myopia, 2- hyperopia, and 3- astigmatism [2]. These disorders can significantly impact a person’s quality of life (QOL) if not corrected optically or surgically [3]. Unlike common eye diseases, such as cataracts and glaucoma, which start in old age, refractive errors may start early in childhood and influence the individual’s activities for many years [4]. RE are prevalent globally, especially in Asia, which, if not corrected, can be considered one of the leading causes of vision loss [5–7]. According to reports from the World Health Organization, 123.7 million people worldwide have visual impairments caused by uncorrected RE [8]. However, once diagnosed, refractive errors can often be easily corrected with spectacles, contact lenses, or refractive surgery. However, failure to recognize it and provide proper correction can cause visual impairment [9].

Visual defects caused by uncorrected RE can adversely affect QOL and limit an individual’s educational, vocational, and social life [10]. Thus, it imposes a significant financial and economic burden on the person and the government [11]. Several researchers have addressed the impact of RE on vision-related QOL. Many studies have been conducted on QOL in people with different refractive errors. These studies usually focus on the differences between the three groups of RE and the effect of various corrections on QOL before and after correction [12, 13]. However, most of these studies ignore very low RE values, which may be considered insignificant. As a result, the QOL of these people is not known. Previous studies have shown that participants with RE below one diopter may have at least two lines of difference between their present visual acuity (VA) and best-corrected VA [3, 9, 14, 15]. Clinical examinations have revealed that individuals with 0.5 diopters of myopia may experience difficulty in seeing details while driving at night or watching TV subtitles. This study compares the QOL among different refractive error subgroups, with a subgroup analysis of low RE, to that of emmetropic individuals.

## Methods

This cross-sectional comparative study was performed on participants with different types of RE in Qom, Iran, in 2021. The inclusion criteria of this study were having an age range of 16 to 35 years and having only one type of refractive error in both eyes (myopia, spherical equivalent (SE) < -0.25 diopters (D), hyperopia, SE > + 0.25 D, astigmatism, cylinder < -0.25 D and emmetropia (-0.25 ≤ SE(D) ≤ + 0.25, cylinder ≥ -0.25) for the control group) [16]. We used the SE for the better spherical eye for myopia and hyperopia. For astigmatism, we used the cylinder values in the eye with less astigmatism, while the spherical values were within the range of -0.25 to + 0.25 in both eyes. We subdivided each group of RE, according to the magnitude, into two subgroups of very low RE (0.50 and 0.75) and significant RE (1.00 ≤) [17]. Correction in this study refers to just using spectacles to correct RE. Uncorrected RE was defined as at least a 2-line difference between presenting and best-corrected VA in the better eye. The participants also needed to have a best-corrected distance VA of 20/20. Exclusion criteria were the use of contact lenses or people who had undergone refractive surgery, the presence of amblyopia, strabismus, any ocular and systemic diseases, history of any ocular surgery, physical and mental impairment, and unwillingness to participate in the study. This study was approved by the Ethics Committee of Shahid Beheshti University of Medical Sciences (IR.SBMU.RETECH.REC.1400.363) and adhered to the tenets of the Declaration of Helsinki.

Before conducting the study, the examiner made sure that all the participants were in good general health and were not taking any medication. Participants were given a detailed explanation of the research’s purpose and then asked to answer the questions. At the beginning of the questionnaire, we also informed the participants that if they use spectacles, they should answer each question while considering the level of vision with their correction. The NEI-VFQ-25 questionnaire was used in this study to evaluate QOL related to vision. Among the questionnaires that examine QOL related to vision and have been translated and validated into Persian, the NEI-VFQ-25 is considered the most reliable and widely used. Asgari et al. translated and validated this questionnaire into Persian in 2011 [18]. The first part of the questionnaire was demographic characteristics, and the next part was divided into 12 subgroups, 11 related to vision and one related to general health. In 2010, Pesudovs et al. conducted a study on the flaws of the NEI VFQ questionnaire [19]. The study highlighted that several questionnaire subscales were not psychometrically appropriate, leading to defective multidimensionality. However, the results improved when the questionnaire was divided into two general parts: visual functioning and socioemotional scales. The researchers recommended using valid long and short forms of the scales to increase the questionnaire’s applicability. The questions dealing with visual functions are included in the long-form visual functioning scales (LFVFS), while those dealing with the emotional and social functioning of the individual are measured by the long-form socioemotional scales (LFSES). Based on the study’s findings, we analyzed data using Rasch analysis on LFVFS and LFSES. In Raschs analysis, the probability of choosing the answer to a particular item depends on the person’s ability and the difficulty of that item. This is taken as a measure of the structure of the responses that one should be satisfied with rather than a simple statistical description of the responses. A person’s ability and item difficulty must refer to the same attribute measured (i.e., visual functioning), so the Rasch model is unidimensional. When data are fit to a Rasch model, measurement estimates are provided on an interval scale, which improves scoring accuracy and tends to remove measurement noise. The unit of measurement in Rasch analysis is logits, which is the natural logarithm of the chance of success in choosing an answer. Tasks with average difficulty are assigned 0 logits. Tasks with difficulty above the average receive a positive logit score, and tasks below the average receive a negative logit score. Therefore, more negative values mean more ease, and more positive values mean more difficulty performing tasks. Individual ability is defined as 0 logits if the respondent has a 50% chance of endorsing an item of average difficulty. A person with a logit score of 2.0 has a 50% chance of selecting an item with a logit difficulty level of 2.0.

Participants underwent a comprehensive and standardized examination, a procedure that included clinical VA testing and a slit lamp examination. VA was determined for each eye separately, with habitual optical correction (glasses or contact lenses) that was measured with the logarithm of the minimum angle of resolution (logMAR). All VA measures were obtained from standardized lighting conditions at 4 m using a logMAR tumbling E chart. The participant’s refractive error without cycloplegic drop was measured with the NIDEK-AR 600 A autorefractor. Then, the results were confirmed using a Heine Beta200 retinoscope, and standardized subjective refraction was performed.

The Winsteps program (version 3.67) was used for Rasch analysis using the Andrich rating scale. Statistical analyses were performed using SPSS 25 software. Mean, standard deviation, median, range, frequency, and percentage were used to analyze the completed questionnaires and describe the data. T tests, Mann‒Whitney tests, chi-squared tests, or Fisher’s exact tests were used to compare the data between the two types of responses to the variable types. ANOVA and Bonferroni comparison were used to compare data between groups. A *P* value less than 0.05 was considered statistically significant.

## Results

**Fig. 1:**
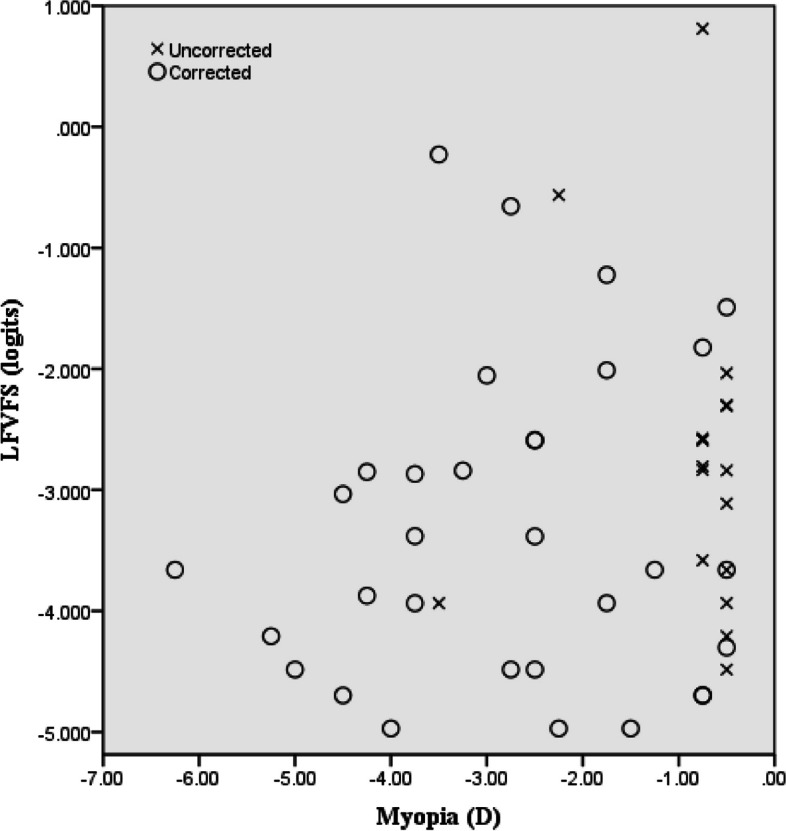
Scatterplot showing the distribution of corrected and uncorrected myopias in different magnitudes and visual function values

**Fig. 2:**
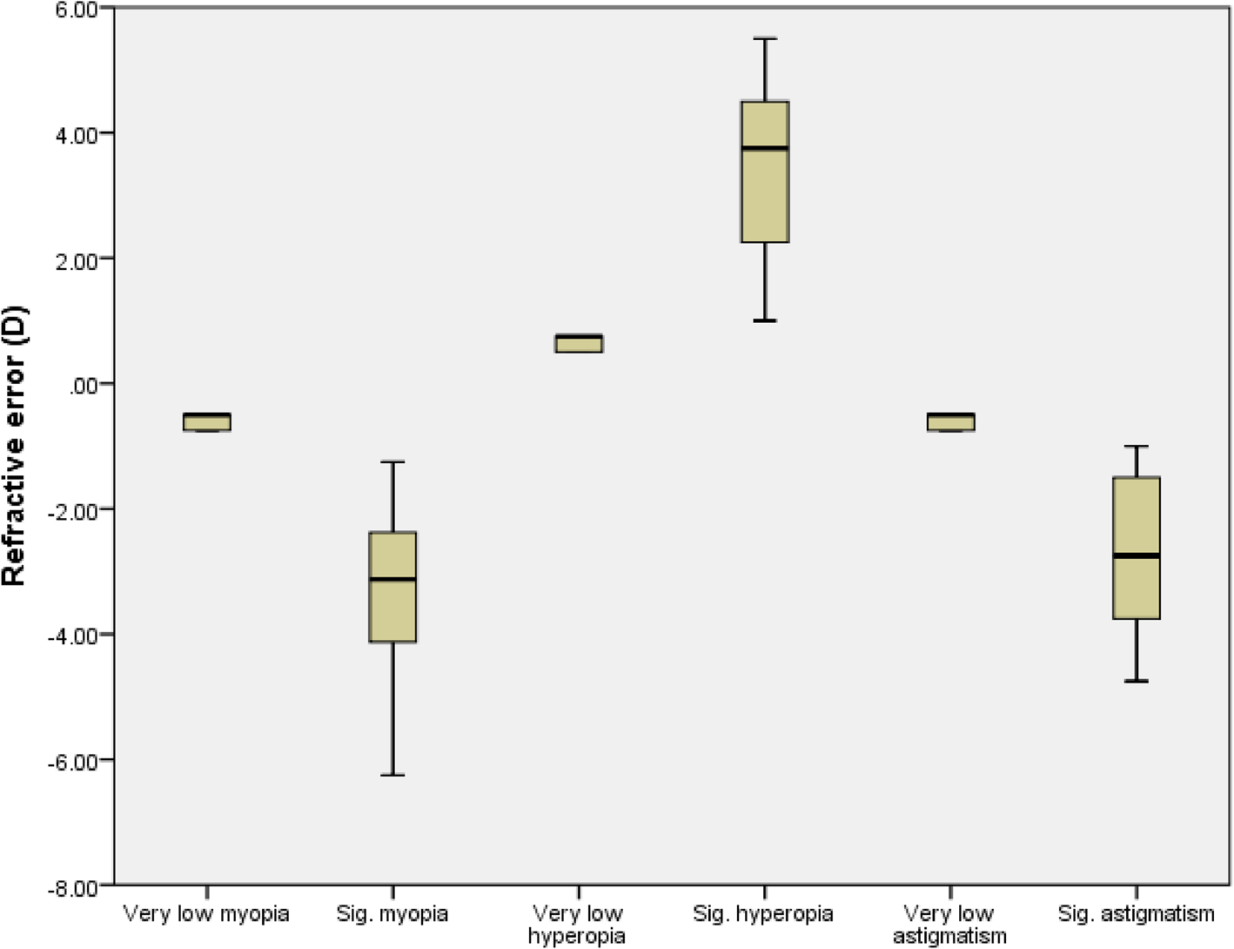
Box-and-Whisker plots for refractive error in different refractive statuses. The boxes represent the 25th to the 75th percentile; the whiskers represent a 1.5 interquartile range outside the boxes. D, diopter; Sig, significant

The study analyzed 200 participants, categorized into four groups of 50 each, consisting of subjects with myopia, hyperopia, astigmatism, and emmetropia, the latter being the control group. The participants’ ages ranged between 15 and 38 years, with an average age of 23.88 ± 5.87 years, and women accounted for approximately 55% of the participants. Table 1 shows the age, number, and percentage of participants in each subgroup. Almost half of the participants with refractive errors (46%) used optical correction. In myopic subgroups, 93% of significant myopes benefited from optical correction, while among very low myopes, only 27% of subjects used optical correction. Notably, this disparity in groups with and without correction was statistically significant within the myopic group. Figure 1 shows the distribution of corrected and uncorrected in different magnitudes of myopia. Conversely, no statistically significant difference was observed across other refractive error categories regarding the utilization of correction between significant and very low values. No significant relationship was observed between the age and gender of the two emmetropes and RE groups and their subgroups. Figure 2 shows the distribution of different values of refractive errors in each subgroup. The mean SE for myopia was − 2.08 ± 1.60 D (range, -6.25 to -0.50 D), while the mean SE for hyperopia was + 2.24 ± 1.59 D (range, + 0.50 to + 5.50 D), and the mean cylindrical value for astigmatism was − 1.82 ± 1.44 D (range, -4.75 to -0.50 D). Table 2 presents the RE and control groups’ average LFVFS and LFSES scores. One-way ANOVA revealed a significant difference in QOL between the RE and control groups (*P* < 0.01). Multiple comparisons were conducted between different groups, and the results showed that people with myopia and hyperopia had a lower QOL in both visual functioning and socioemotional scales than those with emmetropia (*P* < 0.01). However, there was no significant difference between the QOL of participants with astigmatism and the control group.

**Table 1:** Demographic and  clinical characteristics of study participants based on refractive status and magnitude of refractive error

	Myopia	Hyperopia	Astigmatism	Emmetropia	*P* value
Age (mean ± SD)	22.48 ± 5.26	25.12 ± 5.70	24.40 ± 6.00	23.54 ± 6.31	0.131*
Sex N (%)					
Male	17(34)	23(46)	24(48)	26(52)	0.304**
Female	33(66)	27(54)	26(52)	24(48)	
Correction N (%)					
With	33(66)	17(34)	19(38)	2(4)	< 0.001**
Without	17(34)	33(66)	31(62)	48(96)	
Magnitude N (%)					
Significant	28(56)	30(60)	29(58)	-	0.921**
Very low	22(44)	20(40)	21(42)	-	

*N *Number, *SD *Standard deviation

*ANOVA (Multiple comparisons based on Bonferroni)

**Fisher Exact test

**Table 2:** Comparative analysis of vision-related quality of life (QOL) among different refractive status

		QOL (logit)	
		LFVFS Mean ± SD	LFSES Mean ± SD
**Emmetropia**		-4.36 ± 0.65	-2.90 ± 0.31
**Myopia**	**All cases**	-3.12 ± 1.31	-1.96 ± 1.07
	With correction	-3.35 ± 1.28	-2.00 ± 0.97
	Without correction	-2.68 ± 1.28	-1.89 ± 1.27
	*P*-Value	0.480	> 0.999
	**Magnitude**		
	Significant	-3.58 ± 1.15	-2.10 ± 0.99
	Very low	-2.44 ± 1.26	-1.76 ± 1.17
	*P*-Value	< 0.01	> 0.999
**Hyperopia**	**All cases**	-3.47 ± 1.00	-2.29 ± 0.67
	With correction	-3.55 ± 0.97	-2.26 ± 0.44
	Without correction	-3.42 ± 1.03	-2.31 ± 0.77
	*P*-Value	> 0.999	> 0.999
	**Magnitude**		
	Significant	-3.24 ± 0.96	-2.27 ± 0.49
	Very low	-3.62 ± 1.02	-2.30 ± 0.78
	*P*-Value	> 0.999	> 0.999
**Astigmatism**	**All cases**	-3.88 ± 0.90	-2.51 ± 0.77
	With correction	-3.45 ± 0.95	-2.15 ± 0.93
	Without correction	-4.14 ± 0.77	-2.73 ± 0.56
	*P*-Value	0.345	0.169
	**Magnitude**		
	Significant	-3.35 ± 0.89	-1.96 ± 0.99
	Very low	-4.10 ± 0.81	-2.75 ± 0.51
	*P*-Value	0.229	0.015

*QOL *Vision-related quality of life, *LFVFS *Long-form visual functioning scales, *LFSES L*ong-form socioemotional scales, *SD S*tandard deviation

Additionally, it was observed that QOL was significantly lower for participants with myopia than for those with astigmatism (*P* < 0.01). The QOL of corrected and uncorrected myopia, uncorrected hyperopia, and corrected astigmatism was considerably lower than that of emmetropes groups in both visual functioning and socioemotional scales (*P* < 0.01). After multiple comparisons were conducted on different RE magnitudes, QOL in both values of LFVFS and LFSES was less than the control group in all the subgroups, except for very low astigmatism (*P* < 0.01). The comparison subgroups revealed that the QOL in LFVFS with very low myopia was considerably lower than in those with significant myopia, very low hyperopia, and very low astigmatism (*P* < 0.01). On the other hand, participants with very low astigmatism showed better QOL in the LFSES subgroup than those with significant and very low myopia and significant astigmatism (*P* < 0.01).

## Discussion

The NEI-VFQ-25 has been used to investigate QOL in individuals with different types of RE and address those with very low RE who were ignored in previous studies. In the clinical setting, the very low magnitude of refractive error usually leaves clinicians in a dilemma of whether to prescribe or not for these individuals. This research provides insight into QOL and may help clinicians make appropriate decisions. In addition, the QOL of those with spectacle correction was compared to other subgroups, providing further insight into their vision-related QOL.

In this study, uncorrected myopia and hyperopia, reported a worse QOL than the emmetropic individuals. Other studies have also reported lower QOL in uncorrected refractive errors [20–23]. Similar to our findings, another study found that uncorrected myopes had a significantly lower QOL than other groups [14]. Therefore, correcting refractive errors, may improve QOL in myopia and hyperopia groups. In the astigmatism participants, however, our findings suggest that these individuals have visual and social functioning closer to that of emmetropic individuals. This suggests the QOL benefit of correcting astigmatism is less, however, this result may be caused by our participants having medium and low astigmatism values.

In the corrected groups, despite the correction of RE, the level of visual and socioemotional functioning did not reach the level of emmetropic individuals, except for the hyperopia group. These findings could be due to the use of spectacles as the only mode of correction in this study. Spectacles have limitations in terms of optical quality, convenience, and cosmesis. It has been reported that some individuals feel that spectacles are cosmetically unappealing and make them feel less confident or find spectacles cumbersome in sports activities [22]. Other researchers have extensively addressed QOL after correction with a qualitative approach [4]. Therefore, spectacle correction is an imperfect correction. Other modes of correcting refractive error, such as contact lenses and refractive surgery, may be preferred [24].

This study also showed that very low RE individuals had lower visual and social functioning than emmetropic individuals, except for very low astigmatism. The very low myopic group had the worst QOL, which must be considered; most of these people were uncorrected. Surprisingly, the very low hyperopic group did not reach the level of emmetropia. Considering that the refractions were performed without cycloplegia, a higher hyperopic error is expected that might have led to worse QOL. Therefore, correcting very low myopia and hyperopia may be valuable in improving vision-related QOL. Consequently, it is important that low levels of refractive error are not ignored.

The strength of this study is that Rasch analysis was performed on the NEI VFQ-25 to improve the questionnaire into two constructs: visual functioning and socioemotional scales. All the scores obtained were negative, implying that the tasks presented in the questionnaire were easy for the participants. This questionnaire might have not been very specific for measuring quality of life in individuals with refractive error, although it has been used in the past for this purpose. More specific questionnaires are recommended for future studies. Another limitation of this study was that cycloplegic drops were not used. If cycloplegic refraction was performed, a higher refractive errors would have been unmasked with hyperopes. In future studies, it is recommended to use cycloplegic refraction to better estimate the true refractive error.

In conclusion, this study shows that individuals with different types of refractive error have lower QOL than emmetropic individuals. Very low myopia has the worst QOL. Correcting very low refractive errors in participants may improve their quality of life. The shortcomings of spectacles should be discussed. Other interventions, such as contact lenses and refractive surgery, may be offered to those whose visual and socioemotional functioning might be affected by spectacles.

## Data Availability

The datasets used and/or analysed during the current study are available from the corresponding author on reasonable request.

## Abbreviations

QOL: Quality of life
NEI-VFQ-25: 25-Item National Eye Institute Visual Functioning Questionnaire
RE: Refractive error
D: Diopter
SE: Spherical equivalent
VA: Visual acuity
LFVFS: Long-form visual functioning scales
LFSES: Long-form socioemotional scales
